# Possible Neuropathic Pain in Clinical Practice—Review on Selected Diagnostic Tools and Its Further Challenges

**DOI:** 10.3390/diagnostics13010108

**Published:** 2022-12-29

**Authors:** Anna K. Szewczyk, Anna Jamroz-Wiśniewska, Konrad Rejdak

**Affiliations:** 1 Doctoral School, Medical University of Lublin, 20-093 Lublin, Poland; 2 Department of Neurology, Medical University of Lublin, 20-090 Lublin, Poland

**Keywords:** neuropathic pain, questionnaires, neuropathic pain questionnaires, neurological diseases, diagnosis, grading system, screening tools

## Abstract

Background: Neuropathic pain (NeP) is a wide group of conditions provoked by many different causes and with different patterns. The creation of a grading system was intended to determine the level of certainty that the pain is of neuropathic nature. Methods: The aim of this review is to update previously published data on some NeP questionnaires and their measurement properties. The selection of articles is based on the basic neurological units. To assess the usefulness and credibility of the questionnaires, the authors searched for a commonly used measure of reliability, as well as sensitivity and specificity. Results: Studies regarding the usefulness and credibility of questionnaires used in NeP were realized. Different patient cohorts, etiologies and sample sizes, do not allow for an unambiguous comparison of the presented scales; however, all of these studies found good measures of reliability, specificity and sensitivity. Conclusions: NeP tools seem to be beneficial screening instruments that should be utilized by specialists and general practitioners to improve the recognition of “possible” NeP and to determine the epidemiology of this disorder. They have been developed to distinguish perceived pain into neuropathic and non-neuropathic, and, therefore, patients with a mixed pain can still present a diagnostic challenge. Clinical examination and interview play an essential role in the diagnostic process and monitoring, and cannot be neglected.

## 1. Introduction

Neuropathic pain (NeP) is a broad group of conditions provoked by many different causes and with different distributions of pain. It is a pain caused by damage (lesion or disease) of the somatosensory nervous system which can be spontaneous or triggered by sensory stimuli [1]. The true prevalence of NeP is hard to calculate because many disease entities that may be associated with this form of pain are classified according to its etiology, without characterizing the pain as caused by neural damage [2]. Data indicates the population prevalence of NeP between 6.9% and 10% [3], which accounts for up to 25% of individuals with chronic pain (ChP) [4]. This condition may stem from disorders affecting the central (CNS) or peripheral nervous system. This type of pain is typically stubborn and its persistence over 3 months is defined as chronic NeP (ChNeP); however, as diagnostic exceptions allowing the above diagnosis to be made earlier due to clear symptoms, trigeminal neuralgia, pain associated with polyneuropathy caused by type 2 diabetes or central pain after spinal cord injury can be mentioned [5].

Due to the lack of indicative pain biomarkers or pathognomonic features, a conclusive diagnosis of NeP is difficult [4,6]. For this reason, using the mechanical-based approach proposed by the International Association for the Study of Pain (IASP) can help identify this group of patients and to guide further therapeutic decisions. The main purpose of creating the IASP “grading system” was to determine the level of certainty that the pain, existing in individual patients, is of neuropathic nature. This tool can be used in the clinic and in the research, but is not intended to classify the disease. In the grading system, the following three levels have been distinguished: possible NeP, probable NeP and definite NeP. The medical history (a history of relevant neurological lesion or disease) and the information that the patient gives about the pain (pain distribution/distribution neuroanatomically plausible, pain description, various sensory symptoms, aggravating and alleviating factors) he is experiencing, are not pathognomonic but suggestive for NeP. Both of the above elements should be fulfilled to achieve the first level of certainty known as possible NeP. At this level, the questionnaires that contain a combination of several descriptors are helpful to identify patients who may suffer from NeP [7]. The following two different types of questionnaires: screening questionnaires and assessment questionnaires, can be mentioned. The first group has therapeutic implications as being helpful to ameliorate diagnosis, as well as to provide reliable estimates of NeP prevalence in epidemiological studies. The role of assessment questionnaires is to measure NeP symptoms, create phenotypic profiles of NeP symptoms and monitor treatment response. They can also complement screening questionnaires. However, they include neither information derived from clinical or sensory examination, nor negative symptoms. This group contains such questionnaires as the following [8]: the Neuropathic Pain Scale [9], the Neuropathic Pain Symptom Inventory [10], the McGill Short-Form Questionnaire 2 [11] and the Pain Quality Assessment Scale [12]. It is also suggested to include the painDETECT questionnaire (PD-Q) [13] in this group [8,14]. Irrespective of their clinical strength, questionnaires cannot substitute for a proper clinical examination and specialist judgment, as the clinical standard [15].

## 2. Materials and Methods

The aim of this review was to update the previously published data on some NeP screening questionnaires and their measurement properties. Authors decided also to include in the review PD-Q, which can be classified as screening and assessment questionnaire. To identify relevant literature, two independent reviewers screened the available literature based on the PRISMA guidelines [16]. Electronic database searches were conducted until 22 September 2022 on PubMed, MEDLINE and Google Scholar. The studies were published in English, French and Polish. As the search filters were applied the name and/or abbreviation of each NeP screening questionnaire and the word “validation” were used. The literature search was conducted by reviewing abstract and, to identify any additional studies, extended by references lists of included studies and manual searches. Disease entities that go beyond basic neurology (e.g., sickle cell disease, rheumatologic disorders, osteoarthritis), and conducted on minors were excluded from the analysis. To assess the usefulness and credibility of the questionnaires, authors searched for commonly used measures of reliability: Cronbach’s alpha, as well as sensitivity and specificity of the tool. The alpha coefficient estimates reliability and is utilized to measure the internal consistency known as the degree of interaction between items. Its scores vary between 0 and 1 and the ideal measurement value for Cronbach’s alpha is considered acceptable between 0.700 and 0.900 [17,18]. The sensitivity of the screening test is understood as the ability to detect a true positive which reflects a test’s ability to correctly identify people who meet the criterion of interest. The specificity of a test is defined as the test’s ability to correctly identify individuals who do not have the condition of interest, so to detect a true negative [19]. Thus, high specificity and sensitivity allow for inferring a more accurate scale value, therefore they are good enough to use them in surveys.

## 3. Results

### 3.1. The Leeds Assessment of Neuropathic Symptoms and Signs (LANSS) and the Self-Administered Leeds Assessment of Neuropathic Symptoms and Signs (S-LANSS)

The Leeds Assessment of Neuropathic Symptoms and Signs (LANSS) pain scale was published in 2001 in PAIN journal and was originally validated on the English-speaking population. This clinical tool was developed for patients in whom the pain of the neuropathic type predominates and ensures immediate information due to a simple scoring system. The LANSS consists of a pain questionnaire (5-question questionnaire with a dichotomous response YES/NO) and sensory testing (allodynia and altered pin-prick threshold). The pain assessment should be based on the sensations of the last week. The maximum score is 24, with a score of ≥12 indicating a possible neuropathic mechanism. The patients with mixed pain were excluded from the analysis [17].

The LANSS was developed to be a convenient instrument in an epidemiologic survey and large-scale symptom-based research. However, the pin-prick test (part of sensory testing) was given by many researchers as the main objection. For this reason, the authors decided to modify the basic scale to be useful for self-completion. The self-completion LANSS (S-LANSS) contains information about the patient’s current symptoms and signs. For this reason, it may be utilized in postal surveys and clinical settings. For an interview format, optimum cut-off is 10 points, while for unaided 12 points. The S-LANSS contains an image to mark the place of pain with an assessment of its intensity from 0 to 10 and seven questions with a YES/NO response [20].

Studies carried out in Belgium on 2480 patients [21] report differentiation of the types of pain (patterns) between the following two subgroups of patients: with a LANSS score of ≥ 12 and with a LANSS score of < 12. Patients recognized by the instrument with a possible neuropathic mechanism (score ≥ 12) more often complained of a burning sensation, stabbing sensation, electric shock sensation, or dysesthesia. In a provoked pain test, patients with a LANSS of ≥ 12 complained of most conditions/types of allodynia (e.g., to the touch, to contact with clothes, to shaving, etc.) and pin-prick evoked hyperalgesia. Interestingly, in the group which scored ≥ 12 89 percent of patients reported a combination of spontaneous and stimulus-evoked pain versus 36.2% in the second group. Only spontaneous pain was seen in up to 63 percent of patients with LANSS < 12 versus 10.2% in the LANSS ≥ 12 group.

The above-mentioned results (Table 1) prove that both LANSS and S-LANSS are reliable and valid tools to identify the NeP component in ChP patients. The scales have been translated into many foreign languages and are validated and available for use in many countries. The lowest sensitivity results (under 70.0%) of S-LANSS was obtained for the Polish version and of LANSS for the Swedish and Japanese language version, which may be dependent on the small sample size and the special etiology of patient disorders (e.g., spinal cord injury) or different pain descriptions. The Cronbach’s alpha coefficient for all available results, excluding validation for the Persian, Greek and Brazilian-Portuguese version, are within an acceptable range.

### 3.2. Douleur Neuropathique 4 Questions (DN4)

The questionnaire Douleur Neuropathique 4 questions (DN4) is a clinical-administrated tool, which combines the sensory examination performed by a clinician and sensory descriptions. This 10-item questionnaire was intended to be a simple tool with YES (1 point) and NO (0 point) responses. During a patient interview, answers are given to the questions about pain quality (burning, painful cold and electric shocks) and the association of pain with other symptoms in the same area (tingling, pins and needles, numbness and itching). The patient’s examination determines one or more characteristics in the form of hypoesthesia to touch and/or to prick, and the appearance or increase in the pain caused by brushing. A cut-off value of 4/10 results in the highest level of disease detectability with a high sensitivity and specificity [39]. In the original version of DN4, the classification of pain was checked by the two independent physicians and included the following: medical history, physical examination, electromyography and/or imaging examination. All included patients experiencing pain of at least a moderate severity ≥40 mm on the visual analog scale (VAS) [40].

Timmerman H. et al. [40] report in their studies lower validation results in research where participating patients did not undergo initial stratification. Pain intensity may have the main impact on the level of sensitivity and specificity. It is associated with an increase in indicators along with an increase in pain intensity [40,41]. Research shows similar results also in patients with pure neuropathic and mixed (neuropathic and non-neuropathic) pain syndromes [41,42]. In the prospective observational study conducted in seven Canadian academic pain centers, researchers evaluated this research tool for various NeP syndromes and received the highest sensitivity for central NeP and generalized polyneuropathies, while the lowest for trigeminal neuralgia, which proves variable sensitivity depending on the underlying conditions [43]. Likewise, in the study comparing the following four screening tools: DN4, LANSS, S-LANSS and PD-Q, used in diabetic neuropathy, the authors suggest the highest specificity and sensitivity of the first questionnaire. Its additional advantage is the easy and practical implementation in everyday clinical practice, as well as the lack of separate cut-off points. This tool can be also utilized to reduce complications related to the chronicity of the underlying disease (e.g., long-standing diabetes) [44,45]. Based on that questionnaire, patients with diabetic polyneuropathy most commonly describe the pain quality as follows: burning pain, electric shock-like pain and cold pain (71.8%, 38.2% and 36.4%, respectively) [46].

The data cited above (Table 2.) show that DN4 is characterized by both high sensitivity (71.0–100.0%) and specificity (72.4–97.18%). This scale was validated for conditions such as ChNeP, lumbar pain, mixed pain and pain after spinal cord injury. The available data also indicates a quite acceptable value of the reliability expressed as Cronbach’s alpha coefficient of the whole questionnaire.

### 3.3. Neuropathic Pain Questionnaire (NPQ) and Neuropathic Pain Questionnaire—Short Form (NPQ-SF)

The Neuropathic pain questionnaire (NPQ) [58] is composed of 12 items that characterize NeP symptoms, which had previously undergone a gradual discriminant analysis. Those 12 items consist of 10 items that relate to the quality of pain and 2 items that refer to sensitivity changes. Patients are asked to evaluate each item on a scale from 0 to 100 (0—no symptom, 100—worst imaginable severity of symptom); however, it is important to rate the intensity of “usual pain” experienced by the patient. The selection of items was based on the following: positive and negative sensory phenomena, dysesthesia, and paraesthesia, which were described and appeared in NeP patients [40]. The aim of this questionnaire was to offer a standardized assessment of symptoms in an initial screening. Further evaluation of the patient should include a physical and sensory testing examination. Incorporating descriptors important for diagnosis and assessment, this tool can be also useful for patient monitoring [39].

The Neuropathic Pain Questionnaire-Short Form (NPQ-SF) [59] is a shortened version of NPQ, which contains the following three descriptors: tingling pain, numbness and increased pain due to touch. The authors found those three items adequate to divide the patient group into NeP and non-NeP, without statistically significant loss of predictive accuracy. Due to special selection, NPQ-SF includes positive and negative sensory phenomena, but also phenomena suggestive of paraesthesia and dysesthesia. When completing the questionnaire, patients are asked to evaluate each item on a scale from 0 to 100 and write down the result next to it. The results are then multiplied by the coefficient of the discriminant function, added together and then incorporated with a set constant value to create a discriminant function score. A score equal to or greater than 0 indicates the neuropathic nature of pain, while below 0 predicts non-neuropathic pain [60]. Similar calculations, but with other coefficients and constants are created for NPQ [58].

Despite the average sensitivity (50%), NPQ demonstrated the highest specificity (100%) in individuals with pain due to spinal cord injury [35] and 3 discriminants included in the tool, namely, the following: numbness, hypoesthesia to touch and burning pain, as a high fit item. Other authors quote that the fact that NPQ does not include the sensory examination or contains fewer specific items are considered a defect of the questionnaire [26] and may affect the modest accuracy of the questionnaire. After all, it seems that tools based solely on sensory descriptions are distinguished by higher specificity.

Comparative research of the three questionnaires (DN4, S-LANSS and NPQ-SF) conducted by Abolkhair A. et al. [61] showed the excellent diagnostic ability to discriminate between NeP and non-NeP patients of all three questionnaires. NPQ-SF obtained intermediate results of sensitivity and specificity compared to the two other scales. The Spearman rank correlation coefficient between those NeP questionnaires demonstrated a moderate correlation between the tools, perhaps due to similar key verbal descriptors.

The authors of this questionnaire stated that the purpose of its creation was to provide a standardized assessment of symptoms without exclusion of the physical examination with sensory testing. The above-cited data (Table 3.) indicates the moderate sensitivity with fairly good specificity, yet some works suggested a moderate internal consistency of the tool.

### 3.4. PainDETECT Questionnaire (PD-Q)

This questionnaire was published first in 2006 by Freynhagen R. et al. [13]. The purpose of this work was to develop an easy tool to detect NeP components, especially in low back pain since in the study validation, adult patients with various types of chronic low back pain took part. The aim was also to establish a screening tool to reveal NeP components in ChP disorders in order to choose the correct treatment and to establish an easy to administrate and self-reported tool in primary care. The most appropriate scores (the cut-off points) are marked below 12 points and above 19 points, the first one indicating an unlikely neuropathic component, and the second—likely a neuropathic component. Uncertainty remains in the range of 12–19 points when the NeP components can be present.

The PD-Q questionnaire contains four domains. The first domain includes three questions that assess the severity of pain (pain severity at the moment, the strongest pain during the past 4 weeks and the average pain severity during the past 4 weeks) rated from 0 to 10. In the second domain, the patient selects one graph which shows the pain course patterns. The third part requires the identification of the part of the body, where the patient is experiencing pain. If this pain radiates, the direction in which the ailments spread must be marked in the same image. Then, seven questions about pain quality (burning sensation, tingling and pricking sensation, painful light touch, sudden pain attacks, painful cold or heat, numbness, sense of painful pressure) with the following six possible answers to choose from: never, hardly noticed, slightly, moderately, strongly, very strongly, each answer corresponds to a given score from 0 to 5. A final score between −1 and 38 can be achieved. Apart from chronic low back pain, this questionnaire has been used in such disease entities as follows: rheumatoid arthritis, osteoarthritis, fibromyalgia, diverse musculoskeletal conditions where mixed pain predominates, as well as Guillain-Barré syndrome and Charcot-Marie-Tooth type 1A [64,65,66,67,68,69]. The NeP component tends to be overlooked, especially in patients with complaints of musculoskeletal pain [42,70]. For this study group, the work productivity score found a relatively low level of absenteeism (19.3%) with results exceeding 50% for the following: presenteeism, overall work impairment and activity impairment (51.97%, 55.2% and 58.7%, respectively), which may be associated with a high economic burden [71]. Therefore, PD-Q should be also implemented for evaluating pain phenotypes, especially neuropathic-like symptoms (where there seems to be a link between generalized pain and central sensitization symptoms) in patients with heterogeneous musculoskeletal pain [72]. In stable patients, long-term test-retest stability for PD-Q has also been proved, which allows this instrument to be classified as a monitoring tool (assessment questionnaire) in clinical pain trials [14,73].

The table (Table 4) shows that, compared to the original questionnaire, only Hindi, Korean, Italian and Dutch versions exceed 80.0% of sensitivity, while the specificity of all the presented scales, except Korean, Italian and Dutch versions, exceeds 80.0%. It is distinguished by good or very good measure of internal consistency (Cronbach’s alpha coefficient between 0.760 and 0.860). PD-Q has quite good psychometric properties, even for patients with mixed pain mechanisms, and can therefore be considered as a quite good discriminative feature. The differences in sensitivity and specificity results may be due to the specific conditions such as the type of disease entity on which the scale was validated, or the intensity of pain in screened patients (e.g., moderate) [74]. Nonetheless, PD-Q appears to have a unique ability to recognize NeP scores based on the mean pain severity scores (among mild, moderate and severe levels) in patients with NeP [74,75].

### 3.5. Identity Pain Questionnaire (ID-PAIN)

ID-PAIN was developed in a multicenter study, to create easy-to-use, self-administered and useful in primary care screening tools with a low risk of false positive results. Patients with ChP of nociceptive, neuropathic and mixed etiology participated in its creation. Patients experiencing headaches for at least 30 days, as well as patients in unstable medical or psychological conditions or participating simultaneously in another pain study, were excluded from the analysis when creating the tool. Out of the initially proposed 89 items, 6 items identified as meeting the final criteria were chosen (pain like pins and needles, hot/burning pain, numb pain, pain like an electrical shock, pain worse with the touch of clothing or bed sheets and pain limited to joints) to use in the questionnaire. Additionally, on the attached diagram, the patient must mark the painful body area where the pain bothers them the most. Scoring from −1 to 5 was selected, with a higher obtained score associated with NeP or mixed pain with a neuropathic component [84]. The total score of ≥2 points seems to be the best cut-off value to discriminate between patients with or without a neuropathic component [15,85] since the likelihood of pain to be neuropathic is defined as likely (4–5 points), probable (2–3 points), possible (1 point) or improbable (from −1 to 0). Research conducted by Padua L. et al. [86,87] indicates a high association between DN4 and ID-PAIN in NeP identification. For both surveys, the same results were reported by 84% of patients and the discrepancy in the results was affected by the lower degree of pain. Nevertheless, the involvement of a physical examination in DN4 may contribute to a more accurate diagnosis. An interesting report concerning the prediction of mortality in patients with NeP due to type 2 diabetes was presented in a group of 2318 patients. Since ChP was included among the cardiovascular risk factors, ID-PAIN and DN4 were chosen and used for active screening and early detection of peripheral NeP as predictive of vascular event risk and mortality. It is unclear how many patients with diabetes-related polyneuropathy will develop pain, but if the pain is included among the factors affecting the poorer prognosis, it is clear that it should be treated more aggressively [88,89].

Table 5, despite quite good sensitivity and specificity levels (between 77.0%–98.0% and 66.7%–85.0%, respectively), reveals low internal consistency of the whole questionnaire expressed as a Cronbach’s alpha coefficient. However, the high sensitivity; therefore, the low false negative rate, as well as simplicity and brevity, may help general practitioners in the initial screening of the type of pain.

## 4. Discussion

The creation of a screening tool for the grading system in NeP by IASP is intended to help clinicians, neurologists and non-neurologists, to determine the level of certainty of the neuropathic nature of observed pain and to enable the appropriate therapeutic decisions to be made [7]. The inclusion of studies based on the IASP grading system algorithm stands in favor of the mechanism-based approach, which diminishes the heterogeneity of different pain causes and improves clinical trials in evaluating NeP treatments and their efficacy [6]. The introduced changes to the definition oblige the researcher to specify the exact location of the damage to the somatosensory system, otherwise, the lesion is not of neuropathic origin (unless the researcher proves the link between such a structure and the somatosensory processing system). Additionally, “dysfunction” is not treated as an objective assessment, so it was excluded from the criteria [95].

NeP tends to become chronic, then ChNeP is considered to be all states of NeP lasting 3 months or more and persists past the natural healing time; however, some symptoms allow it to be diagnosed earlier [5,96]. Researchers in NeP and ChP report structural and functional changes in the areas that were associated with the modulation and perception of pain. Both types of pain trigger central and peripheral mechanisms and thus functional and signal changes at the cellular and receptor levels, nociceptive transmission at the dorsal horn, or modulation of the nociceptive signaling in the spinal cord [97]. In ChP, both cell-mediates and humoral immunity may be suppressed, as well as gene expression changes may occur. It seems that the application of the correct treatment may at least partially reverse those alterations [98].

The negative impact of ChP on the quality of life is well known [21], especially on misdiagnosed or underdiagnosed and wrongly treated individuals [99]. This chronic condition negatively affects humans’ lives and has an unfavorable impact on many aspects of the patient’s life, inter alia, on family life, work and physical activity, or self-care and self-esteem. In this case, the elements of the vicious circle include physical disability, psychological distress and constant pain [100]. An additional factor, increasing current pain complaints, is sleep disturbance or poor sleep quality. Patients experiencing pain suffer from sleep deprivation generated by difficulty in initiating and maintaining sleep. Some research also suggests complex neurobiological correlations between sleep disorders, ChP and depression [101,102,103]. Perhaps we should talk about the bidirectional nature of the disorder while psychological factors may also incline individuals to ChP [99]. Maybe in the future, patients will be assessed according to their sensory loss phenotype as a response to personalized medicine to propose the most beneficial treatment possible for emotional well-being [104]. An additional facilitation in primary health care may be the application of electronic equipment with electronic rating scales, which, with the help of the appropriate software, will give the same results, as the conventional paper questionnaire [105]. Nevertheless, patients with cognitive impairment or visual and hearing disorders will remain problematic, which results in underdiagnosis [106].

Estimation of NeP prevalence was undertaken with the application of the above questionnaires. In Europe, the reported data is almost 7% (DN4, S-LANSS) [107,108,109], in the US for probable NeP may suffer even 10% of the population (PD-Q) [110], so it is a large-scale problem. To increase the reliability of obtained results some researchers also used two screening tools (e.g., DN4 and PD-Q) [111]. Moreover, it is certain that specialists in various fields will meet patients with uncontrolled or not properly controlled (chronic) NeP [112]. For this reason, it is so important to correctly diagnose them, determine the further course of action and initiate optimal treatment. In elderly patients, the prevalence of NeP can even reach 32% [113]; however, due to comorbidities or fear, there is no conclusive data because patients often under report any discomfort. NeP questionnaires may help them to “visualize” their ailments. They will also require a multidisciplinary team (medical, psychological, social and rehabilitation), to manage their symptoms, support their everyday life and the health effects of polypharmacy [106].

Using different questionnaires for international research studies requires obtaining a language version of this tool. Additionally, they should measure the same concepts and be easy to understand by patients and clinicians. The goal of linguistic validation is to obtain a conceptually equivalent text and not necessarily a literal translation [114]. The validation of questionnaires may be adversely affected by situations where validation is conducted in a research setting, improper selection of the patients’ cohort (should be comparable to the patients/physicians for whom the screening tool is intended) and exclusion of patients (pre-stratification) with mixed pain, which will lead to a non-clinical situation [40]. Although, screening tools for NeP have great potential for use in epidemiological and clinical research, but their limits cannot be forgotten. Those tools may facilitate the guidance of diagnostics for non-specialists nevertheless, they fail to discern 10-20% of patients with clinically diagnosed NeP [8,15]. In the systematic review conducted by Mathieson S. et al. [115], the authors report the highest measurement properties of the original version DN4 and NPQ. The original questionnaires benefit over the language versions, perhaps due to intercultural differences. The research analyzed often excluded patients with mixed pain or based only on a “specific” pain population, which probably influenced the sensitivity and specificity of the questionnaires and consequently reduce their usefulness in clinical practice. The authors encourage investigators to re-evaluate the screening tools by using the new (IASP) definition of NeP [3] to revise the measurement properties. The diagnostic accuracy of the questionnaires remains limited for neck/upper limb pain [116]. It may be advisable to refer patients with the above symptoms to specialized units or to assume decision support in primary health care to provide the appropriate care. Two clinicians would appear to be desirable in the grading evaluation to avoid clinical bias due to differences in the interpretation of sensory changes and imaging results. For experienced clinicians, the revised NeP classification can be reliably applied, yet the authors propose its further improvement, to include other clinical findings such as motor or reflex changes that will correspond with a relevant disease or lesion [117].

## 5. Future Is Now

Patients with tumor-related cancer pain or chemotherapy-induced pain will be an increasing group of responders with sensory disturbances in outpatient clinics or hospitals. Over half of the patients with advanced cancer and one-third of the patients receiving anti-cancer treatment will feel pain, especially as cancer survival rates are increasing [118]. To explain the etiology of pain in neuropathic cancer pain, lesion or lesions should be identified. Nonetheless, standardized methods of defining are still not available. Hitherto various methods have been used, such as a combination of a neurological lesions and specific symptoms, searching for disease-induced damage to the CNS or the screening tools application. Nevertheless, it seems uncommon for cancer pain to be caused solely by damage to the CNS, which is why a standardized approach to the assessment is needed [119]. It is probable that the NeP symptoms in oncological patients begin with common symptoms such as pins and needles, tingling and electric shocks [120].

A French study [121] reports chronic pain in up to 35% of oncological patients, with 20–25% of patients suffering from the neuropathic component among them. This type was more frequent in breast, lung or head/neck cancers. Additionally, by using DN4, researchers indicated an increase in time number of patients with pain, 12–20% of the patients had developed pain by the 3-month visit, while by the 6-month visit—up to 28% of responders. In terminally ill patients, the prevalence of NeP is estimated at 30.6% [122]. A cross-sectional study was conducted on patients with breast cancer, by using ID-PAIN and S-LANSS. The authors found that 67% of patients with an S-LANSS score of ≥ 12 had a positive ID-PAIN (score of ≥ 2), as well as 93% of patients with a negative S-LANSS, were also negative in ID-PAIN. These results indicate that ID-PAIN in breast cancer patients can be treated as sensitive, yet less specific than S-LANSS. Differences between different published studies regarding the prevalence of NeP in breast cancer survivors may result from the use of different assessment tools, which may be indicative of problematic NeP evaluation for this group of responders [87,123]. A survey using PD-Q and S-LANSS shows their low credibility to identify the neuropathic component of mixed pain, especially if it concerns moderate to severe cancer pain [124], the same inconvenience was depicted using DN4 and PD-Q in a cross-sectional study [125]. This is in contradiction with the research carried out by Pérez C et al. [126], where authors state that both, LANSS and DN4 were advantageous in the early detection of patients at risk (DN4) and ruling out NeP in patients with complex pain conditions (LANSS). These differences may result from the use of other questionnaires for studies, but also from the difference between the groups of respondents.

A systematic literature search which recruited 2301 cancer patients evaluated the methodological quality of NeP assessment tools. A large variation in sensitivity and high level of specificity across the analyzed questionnaires (LANSS, DN4 and PD-Q) but also more frequent mixed pain mechanisms in cancer or postsurgical patients influence the authors’ careful approach to their use. This also applies to the IASP “grading system”, which was not designed to identify NeP within mixed pain syndromes [127]. The diagnostic difficulty of patients with mixed pain for physicians in primary care is compounded by the lack of formalized screening or diagnostic tools. However, some validated NeP questionnaires can detect the presence of this component (S-LANSS, DN4 and PD-Q). Even so, diagnosis of mixed pain requires clinical judgment based on clinical evaluation after taking a detailed history and thorough physical examination, which remains a challenge for a clinician with little experience or no training in pain assessment [128,129]. It follows that future research is needed, in particular, to standardize the clinical diagnosis and way of further proceedings for non-pain specialists.

Post-COVID pain following viral infection is also becoming a growing problem. It is assumed that some patients infected with COVID-19 as a result of the post-viral immune syndrome will develop NeP within weeks or months. An additional problem may determine patients with exacerbation or deterioration of existing pain [130]. A telephone survey was conducted in north-western Turkey [131] using NPQ. Up to 25% of interviewees reported NeP symptoms, in the form of the following: numbness, burning pain and squeezing. Spanish survey applied S-LANSS in COVID-19 survivors [132], 19 patients out of 77 (24.6%) included in the study obtained a score of ≥12, indicating a possible neuropathic mechanism. Similar results (24.4%) for NeP prevalence were obtained in a study with the use of PD-Q and DN4 [133]. The authors estimated also the prevalence of ChP in post-COVID patients at 63.3%. S-LANSS and PD-Q were used in a study conducted by Fernández-de-las-Peñas C. et al. [134], which included 146 participants. According to S-LANSS, 26% of patients had NeP symptoms, as opposed to 12.2% of the patients surveyed with PD-Q. Thirteen patients (8.8%) obtained in PD-Q score between 12 and 18 points, which signifies ambiguous NeP origin. The authors explained this difference by different component assessments of both questionnaires.

Nevertheless, it is not yet possible to evaluate the prevalence and clinical characteristics of post-COVID pain. Further research is required to determine the best way to recognize and treat these patients, the long COVID may prove to be an additional challenge.

According to the comparison prepared by Bennett MI. et al. [77], all five presented above questionnaires (LANSS, DN4, NPQ, ID-PAIN and PD-Q) include the following symptoms:

− Picking, tingling, pins and needles;
− Electric shock or shooting;
− Hot or burnings.

Four of them (namely, DN4, NPQ, ID-PAIN and PD-Q) check numbness, and all except DN4 search for pain evoked by light touch. Only ID-PAIN contains the question “is the pain limited to your joints?” used to identify non-neuropathic pain. Clinical examination is needed when filling DN4 and LANSS. Evaluation of brush allodynia and raised pinprick threshold is possible in both cases, while raised soft touch threshold with DN4 use. Despite the differences in tool development, the authors [135] rely on their reliability and the validity of their approach. Reaching a consensus on which tool is the most appropriate in the particular context will depend on the use of scales by other specialists and researchers, as well as the validation of tools in other languages and cultures. This should apply to primary care patients with different components of pain, patients with mixed pain due to cancer or chemotherapy, as well as individuals with postsurgical pain [136,137]. The advantages and disadvantages as well as the application of questionnaires have been well and thoroughly described in the paper of RCW Jones 3rd and MM Backonja [138].

## 6. Limitations

This review has several limitations. As was already mentioned, screening questionnaires may be useful to improve recognition of NeP but provide no information about the clinical history and may lead to over- or underdiagnosis. Variable underlying conditions may lead to bias in terms of estimating screening tools performance. Among them are different pain syndromes, inclusion or exclusion of patients with mixed-type pain, as well as the different proportion of patients across the studies can be mentioned. These may result in reduced sensitivity and specificity of the validated scale.

## 7. Results and Conclusions

Presented above screening tools used in NeP (LANSS, S-LANNS, DN4, NPQ, NPQ-SF, ID-PAIN and PD-Q) are characterized by quite good or good measurement properties and were validated for different pain conditions. Moreover, available results of internal consistency for almost all presented results are within an acceptable range. From a practical perspective, the simplicity of the survey, when used systematically, makes it possible to identify a large proportion of unrecognized “possible” NeP patients. Such a procedure, in the case of a nonspecialist or family doctor, allows shortening the time of making the correct diagnosis and starting treatment by a qualified specialist. An interview and neurological examination of the patient are needed to assess the potential cause of NeP. Increased emphasis on the search for this disease entity may also significantly affect epidemiological research to correctly estimate the overall prevalence of (chronic) pain with neuropathic characteristics. Assessment questionnaires have their role in monitoring the patient’s treatment, but also can be used to complement screening questionnaires.

However, the possibility of misdiagnosis must be taken into account. An inaccurate diagnosis is made in up to 20% of cases, in particular, in mixed pain conditions. Additionally, questionnaires that do not include sensory examination are considered by some researchers, as defective and may affect their modest accuracy. Nevertheless, it would be difficult to require a full neurological examination performed in primary care. Instead, verified, validated, easy-to-use and/or self-reported screening tools may ensure faster referral of the patient to targeted tests.

Despite advantages and disadvantages, created and previously validated tools seems to be a beneficial screening instrument that should be utilized by specialists and general practitioners to improve recognition of “possible” NeP and to determine the epidemiology of this disorder. All the questionnaires were translated into many foreign languages; therefore, they can be easily understood by patients and clinicians and utilized as a differentiating tool in outpatient clinic or during hospitalization. In case of uncertainty, it is possible to use two different surveys, which can be selected depending on underlying conditions or comorbidities. Questionnaires have been developed to distinguish perceived pain as neuropathic and non-neuropathic, mixed pain can therefore still present diagnostic problems. Further research and determination of diagnostic standards should be crucial in this broad group of conditions. Moreover, the evaluation of screening tools in a “new context” is needed to estimate their effectiveness and to standardize the diagnostic approach. It needs to be emphasized that correct clinical examination has an essential role in the further therapeutic process and patient monitoring. Nevertheless, the concordance between screening tool outcomes and clinical diagnosis makes the questionnaire practical and can be considered the first step in identifying potential NeP cases.

Research on NeP should continue as NeP tends to be a serious clinical problem under development, while its complications will probably become a major healthcare problem worldwide. A large group of patients with a different types of pain, including NeP, will appear in our outpatient and/or hospital practice, so it is important to diagnose them quickly, transfer them for further observation, or just start proper treatment. This problem seems to be much bigger when taking into account the undesirable ailments caused by pain, (such as reduced sleep quality, polypharmacy and the impact on social life) and the fact that ChP has been included as one of the cardiovascular disease risk factors, which is significant for an aging population. Personalized medicine is desirable for the future development of this entity.

## Figures and Tables

**Table 1:** List of articles with LANSS/S-LANSS validation.

Authors, Year	Population/Version	LANSS/S-LANSS	Number of Patients	Sensitivity	Specificity	Cronbach’s Alpha Coefficient of the Whole Questionnaire
Bennett M. (2001) [17]	Original English Version	LANSS	40	85.0%	80.0%	0.740
Bennett M. et al., (2005) [20]	Original English Version	S-LANSS	200	74.0%	76.0%	0.760
Spanos K. et al., (2015) [22]	Greek Version	LANSS	70	94.29%	88.57%	0.895
Cnotliwy M. et al., (2016) [23]	Polish Version	S-LANSS	101	62.0%	77.0%	NA
Koc R. et al., (2010) [24]	Turkish Version	S-LANSS	244	72.3%	80.4%	0.740
Türkel Y. et al., (2014) [25]	Northern Turkey Version	LANSS	148	98.0%	97.0%	0.961
Migliore A. (2021) [26]	Italian Version	LANSS	100	87.0%	72.0%	0.760
Park C. et al., (2015) [27]	Korean Version	LANSS	213	72.6%	98.0%	0.815
Barbosa M. et al., (2014) [28]	Portuguese population	LANSS	165	89.0%	74.0%	0.780
Isomura T. et al., (2017) [29]	Japanese version	LANSS	59	63.3%	93.1%	NA
López-de-Uralde-Villanueva I. et al., (2018) [30]	Spanish Version	S-LANSS	321	88.7%	76.6%	0.710
Hamdan A. et al., (2014) [31]	Spanish version	LANSS	192	80.17%	100.0%	NA
Li J. et al., (2012) [32]	Chinese version/Mandarin language	LANSS	140	80.0%	97.1%	0.827
Saghaeian S. et al., (2020) [33]	Persian version	LANSS	206	88.12%	76.19%	0.640
Saghaeian S. et al., (2020) [33]	Persian version	S-LANSS	206	83.17%	95.24%	0.610
Unal-Cevik I. et al., (2010) [34]	Turkish version	LANSS	180	70.2%	96.6%	NA
Hallström H. et al., (2011) [35]	Swedish version	LANSS	40	35.7%	100.0%	NA
Schestatsky P. et al., (2011) [36]	Brazilian-Portuguese version	LANSS	90	NA	NA	0.670
Batistaki C. et al., (2016) [37]	Greek version	LANSS	200	82.76%	95.24%	0.650
Batistaki C. et al., (2016) [37]	Greek version	S-LANSS	200	86.21%	95.24%	0.670
Ezlahaf r. et al., (2013) [38]	Arabic version for use in a Libyan Population	S-LANSS	104	NA	NA	0.720

NA—not applicable.

**Table 2:** List of articles with DN4 validation.

Authors, Year	Population/Version	Number of Patients	Sensitivity	Specificity	Population Type	Cronbach’s Alpha Coefficient of the Whole Questionnaire
Bouhassira D. et al., (2005) [39]	Original version (French, English)	160	82.9%	89.9%	ChNeP and non-NeP, VAS ≥ 40 mm	NA
Sykioti P. et al. (2015) [47]	Greek version	237	93.0%	78.0%	ChNeP, nociceptive and mixed pain, VAS ≥ 5	0.650
Madani S. et al. (2014) [48]	Persian version (Farsi language)	175	90.0%	95.0%	ChNeP and non-NeP, VAS ≥ 40 mm	0.852
Perez C. et al. (2007) [41]	Spanish version	158	79.8%	78.0%	ChNeP, mixed pain and non-NeP	0.710
Hamdan A. et al., (2014) [31]	Spanish version	192	95.04%	97.18%	ChNeP and non-NeP, VAS ≥ 40 mm	NA
Wang Y. et al., (2019) [49]	Taiwan version/Mandarin Chinese language	330	77.0%	78.0%	ChNeP, mixed pain and non-NeP	0.700
Chatila N. et al., (2017) [50]	Arabic version	195	93.0%	95.8%	ChNeP and non-NeP	NA
Van Seventer R. et al., (2013) [51]	Dutch version	269	74.0%	79.0%	ChNeP and non-NeP, NRS ≥ 5	NA
Saxena A. et al., (2021) [52]	Hindi version	285	78.0%	76.0%	ChNeP and non-NeP, VAS ≥ 4 cm	0.820
Terkawi A. et al., (2017) [53]	Arabic version	142	88.31%	74.47%	NeP and non-NeP	0.670
Santos J. et al., (2010) [54]	Portuguese version	101	100.0%	93.2%	ChNeP and nociceptive pain, NRS ≥ 4	0.760
Unal-Cevik I. et al., (2010) [34]	Turkish version	180	95.0%	96.6%	NeP and non-NeP	0.970
Kim H. et al., (2016) [55]	Korean version	83	87.1%	94.1%	Nociceptive pain and NeP in lumbar or lumbar-radicular pain, VAS ≥ 4	0.819
Hallström H. et al., (2011) [35]	Swedish version	40	82.9%	75.0%	Spinal cord injury	NA
Matsuki Y. et al., (2018) [56]	Japanese version	187	71.0%	92.0%	NeP and non-NeP	0.604
Harifi G. et al., (2011) [57]	Moroccan Arabic Dialect version	170	89.4%	72.4%	NeP and non-NeP	0.630

NA—not applicable, VAS—visual analogue scale, NRS—numerical rating scale, ChNeP—chronic neuropathic pain, NeP—neuropathic pain, non-NeP—non-neuropathic pain.

**Table 3:** List of articles with NPQ/NPQ-SF validation.

Authors, Year	Population/Version	Version NPQ vs. NPQ-SF	Number of Patients	Sensitivity	Specificity	Cronbach’s Alpha Coefficient of the Whole Questionnaire
Krause S., Backonja M. (2003) [58]	Original version	NPQ	382	66.6%	74.4%	NA
Backonja M., Krause S. (2003) [59]	Original version	NPQ-SF	278	64.5%,	78.6%	NA
Hallström H. et al., (2011) [35]	Swedish version	NPQ	40	50.0%	100.0%	NA
Li J. et al., (2012) [32]	Chinese version /Mandarin language	NPQ	140	52.9%	91.4%	0.809
Yurdakul O. et al., (2019) [62]	Turkish version	NPQ	101	NA	NA	0.840
Yurdakul O. et al., (2019) [62]	Turkish version	NPQ-SF	101	NA	NA	0.670
Shafiee E. et al., (2021) [63]	Persian version	NPQ	101	84.0%	64.0%	0.810
Terkawi A. et al., (2019) [60]	Arabic version	NPQ-SF	142	71.0%	71.0%	0.450

NA—not applicable.

**Table 4:** List of articles with PD-Q validation.

Authors, Year	Population/Version	Number of Patients	Sensitivity	Specificity	Population Type	Cronbach’s Alpha Coefficient of the Whole Questionnaire
Freynhagen R. et al., (2006) [13]	Original version (German)	392	84.0%	84.0%	Chronic low back pain	0.830
Ghamkhar L. et al., (2021) [76]	Persian version	150	74.70%	98.51%	ChP/low back pain, knee pain, neck pain and shoulder pain	0.760
De Andrés J. et al., (2012) [77]	Spanish version	221	75.0%	84.0%	ChP/NeP, non-NeP, mixed pain	0.860
Alkan H. et al., (2013) [78]	Turkish version	240	77.5%	82.5%	NeP, mixed pain and nociceptive pain	0.810
Gudala K. et al., (2017) [79]	Hindi version	160	82.5%	91.2%	NeP and non-NeP	0.830
Abu-Shaheen A. et al., (2018) [80]	Arabic version	375	67.3%	81.1%	NeP and nociceptive pain	0.764
Sung J. et al., (2017) [81]	Korean version	232	95.4%	73.8%	NeP, nociceptive pain and mixed pain	0.804
Timmerman H. et al., (2018) [82]	Dutch version	291	80.0%	55.0%	ChP/low back with leg pain, neck-shoulder-arm pain or a suspected peripheral nerve damage pain	NA
Matsubayashi Y. et al., (2013) [83]	Japanese version	122	NA	NA	NeP and nociceptive pain	0.780
Rio J. et al., (2022) [65]	Brazilian-Portuguese version	30	NA	NA	Musculoskeletal pain	0.830
Hallström H. et al., (2011) [35]	Swedish version	40	67.9%	83.0%	Spinal cord injury	0.830
Migliore A. et al., (2020) [26]	Italian version	100	85.0%	75.0%	Trigeminal or postherpetic neuralgia as NeP	0.800

NA—not applicable, ChP—chronic pain, NeP—neuropathic pain, non-NeP—non-neuropathic pain.

**Table 5:** List of articles with ID-PAIN validation.

Authors, Year	Population/Version	Number of Patients	Cut-off Value	Sensitivity	Specificity	Cronbach’s Alpha Coefficient of the Whole Questionnaire
Portenoy R. (2006) [84]	Original version	308	NA	NA	NA	NA
Khodabandeh B. et al., (2022) [85]	Persian version	90	≥2	98.0%	79.0%	0.470
Abu-Shaheen A. et al., (2018) [90]	Arabic version	375	≥2	84.3%	66.7%	0.506/0.531
Yang CC. et al., (2018) [91]	Taiwan version	317	≥2	77.0%	74.0%	0.600
Kitisomprayoonkul W. (2011) [92]	Thai version	100	≥2	83.0%	80.0%	0.318
Gálvez R. et al., (2008) [93]	Spanish version	283	≥3	81.0%	84.0%	NA
Li J. et al., (2012) [32]	Chinese version	140	≥1	97.1%	72.9%	0.755
Uzunkulaoğlu A. et al., (2019) [94]	Turkish version	194	≥2	77.2%	85.0%	0.701
Padua L. et al., (2013) [86]	Italian version	392	NA	78.0%	74.0%	NA

NA—not applicable.

## Data Availability

Not applicable.
